# Construct Validity of the Holistic Complementary and Alternative Medicines Questionnaire (HCAMQ)—An Investigation Using Modern Psychometric Approaches

**DOI:** 10.1093/ecam/nep141

**Published:** 2011-01-11

**Authors:** Paula Kersten, P. J. White, A. Tennant

**Affiliations:** ^1^School of Health Sciences, University of Southampton, Southampton SO17 1BJ, UK; ^2^Academic Unit of Musculoskeletal and Rehabilitation Medicine, University of Leeds, Leeds LS2 9JT, UK

## Abstract

The scientific basis of efficacy studies of complementary medicine requires the availability of validated measures. The Holistic Complementary and Alternative Medicine Questionnaire (HCAMQ) is one such measure. This article aimed to examine its construct validity, using a modern psychometric approach. The HCAMQ was completed by 221 patients (mean age 66.8, SD 8.29, 58% females) with chronic stable pain predominantly from a single joint (hip or knee) of mechanical origin, waiting for a hip (40%) or knee (60%) joint replacement, on enrolment in a study investigating the effects of acupuncture and placebo controls. The HCAMQ contains a Holistic Health (HH) Subscale (five items) and a CAM subscale (six items). Validity of the subscales was tested using Cronbach alpha's, factor analysis, Mokken scaling and Rasch analysis, which did not support the original two-factor structure of the scale. A five-item HH subscale and a four-item CAM subscale (worded in a negative direction) fitted the Rasch model and were unidimensional (χ2 = 8.44, *P* = 0.39, PSI = 0.69 versus χ2 = 17.33, *P* = 0.03, PSI = 0.77). Two CAM items (worded in the positive direction) had significant misfit. In conclusion, we have shown that the original two-factor structure of the HCAMQ could not be supported but that two valid shortened subscales can be used, one for HH Beliefs (four-item HH), and the other for CAM Beliefs (four-item CAM). It is recommended that consideration is given to rewording the two discarded positively worded CAM questions to enhance construct validity.

## 1. Introduction

The prevalence of osteoarthritis (OA) has been reported to be as high as 8.5 million people in the UK [1] and many patients experience considerable comorbidity [2, 3]. While the debate about the efficacy of complementary medicine continues, its use among OA patients is widespread as a primary therapy, or secondary to traditional medicine [4, 5]. There is also increased recognition that rigorous studies are required if complementary medicine is to be taken seriously by those working in public health services, and those who fund healthcare [4, 6]. Part of the scientific basis of such studies would be the availability of validated measures to operationalize the bio-psychosocial model within which such an evaluation is likely to be set. Such a model would include not only measures of impairment and activity limitations, but also key mediating factors which might be expected to influence outcome. These factors may include aspects of holistic health beliefs as well as attitudes toward complementary medicine.

Few scales are available for this purpose at the present time. One is the Attitudes Toward Alternative Medicine Scale (AAMS) [7]. The AAMS was examined for validity as part of a randomized, double-blind, placebo-controlled trial with 327 patients allergic to house dust mites in Southern England [8]. A factor analysis showed a valid two-factor structure in which one factor included attitudes toward complementary and alternative medicine (CAM) and the second a belief that the body varies in terms of “a healthy balance”. Another scale is the Holistic Complementary and Alternative Medicine Questionnaire (HCAMQ) developed by Hyland et al. [9]. This includes six questions from the AAMS and six questions from the Holistic Health (HH) Beliefs Questionnaire [9]. This validation study included 50 patients attending out-patients rheumatology and 50 patients attending the Centre for the Study of Complementary Medicine. Patients were included if they were >18 years of age and fluent in English. All items were scored in the pro-CAM and pro-HH direction (where a lower score reflects a pro-attitude toward CAMs and HH). Two factors were extracted using Principal Axis factoring with oblimin rotation (factor loadings >0.30 deemed significant), the correlation between the two factors was 0.26; all six CAM questions loaded highly on the first factor. Five of the HH items loaded >0.30, one had an unsatisfactory loading. The resulting HCAMQ consists therefore of two subscales: the CAM subscale consisting of six items and the HH subscale consisting of five items (Table 1). The correlation between the two subscales led the authors to conclude that there is a higher order construct at play and that therefore a total HCAMQ can be used. Cronbach alpha coefficients were acceptable (CAM subscale 0.83, HH five-item subscale 0.75), confirming internal consistency. The CAM subscale was able to discriminate between the two groups of patients, but not the HH subscale. Test-retest reliability of the scale was satisfactory as measured by the ICC (CAM subscale 0.82, HH subscale 0.77). Discriminant validity for the CAM was demonstrated by a difference in scores in the two patient groups (although the authors did comment this could have arisen due to a hawthorn effect in the group attending the CAM clinic). Further, convergent validity was found between reported vitamin use and both of the HCAMQ subscales and between antibiotics use and the CAM subscale.

The HCAMQ was further examined during a recent survey among 448 healthy people attending a primary care center for healthcare services in Turkey [10]. Cronbach alphas were reported as good (internal consistency) for the total HCAMQ (0.72), CAM subscale (0.62) and the HH subscale (0.60). The two-factor structure of the HCAMQ was confirmed with a principal axis factoring with oblimin rotation. Correlation between the two factors was 0.47 which led the author to conclude that a total score can be calculated.

Despite evidence from traditional approaches, recent advances in modern psychometric techniques have led to further examination of existing scales to test their reliability and validity against rigorous standards of measurement [11]. The HCAMQ, a promising scale, has not been examined using these approaches. Consequently this article aims to examine the construct validity of the HCAMQ, both subscales and a total score, using a modern psychometric approach, in order to build on the existing preliminary evidence for the usefulness and appropriateness of the scale in clinical practice and research.

## 2. Methods

### 2.1. Patients and Data Recording

A study which aimed to investigate the relative effects of acupuncture and different currently used acupuncture placebo controls recruited 221 patients. All had chronic stable pain predominantly from a single joint (hip or knee) of mechanical origin and were waiting for a hip (40%) or knee (60%) joint replacement, scored a minimum of 30 on a 100 mm VAS scale for pain averaged over a one week baseline, and were not on active treatment (apart from their normal analgesia). Those with serious comorbidity, pregnant, prolonged or current steroid use or waiting for a joint revision were excluded.

Among a range of data recorded, responses to the HCAMQ were obtained. Consisting of 11 questions, they are divided into the HH Subscale which contains five items and the CAM subscale containing six items. Responses were recorded on a six-item Likert-type scale, ranging from strongly agree to strongly disagree. As some of the questions are worded positively and others negatively reversing scores for some items is required. In the original HCAMQ article the data were scored so that a low score reflected a pro-attitude toward HH and CAMS [9]. However, in the current study the scores were reversed so that a high score reflected a pro-attitude toward HH and CAMS as this provided greater clarity in interpretation alongside other scales used. The HCAMQ was self-completed by patients on entry into the study.

### 2.2. Data Analysis

Reliability of the HCAMQ was determined by Cronbach alpha of the subscales, and deemed acceptable if >0.70 [12]. Within the Rasch analysis (see below) reliability was also measured through a Person Separation Index [13], equivalent to alpha, but, because it is based upon a linear estimate of person ability, rather than the raw score, it can be calculated where missing values are present.

Initially, data were examined by a factor analysis to confirm the 2D structure of the scale, with parallel analysis to determine the significant eigenvalues [14]. The parallel analysis creates 100 sets of random data, of the same size as our own data, and average eigenvalues for these samples are calculated. Each subsequent eigenvalue from our data is then compared with the average eigenvalue generated in the parallel analysis and if our own eigenvalues are found to be greater they are deemed significant. The data were also subjected to Mokken scaling to determine if there existed a non-parametric probabilistic Guttman-style relationship in the data [15–17]. The latter would determine if the set of items made a valid ordinal scale [18]. Acceptability of the probabilistic relationship was determined by a Loevinger *H*-coefficient >0.3 for individual items and the scale as a whole. The procedure uses a distinct approach in that unlike factor analysis or Rasch analysis (both of which are parametric procedures) which start with a predetermined set of items supposedly belonging to a single (or multiple) construct, Mokken scaling works “bottom-up” by starting with the two items that have the strongest correlation, and then adding further items which satisfy the Loevinger level given above. An attempt to construct a second (and subsequent) scale is made when there is more than one item remaining. Further details of the process can be found elsewhere [18, 19].

Data from the subscales were then fitted to the Rasch measurement model [20]. The process of Rasch analysis is also described in detail elsewhere [11, 21]. Briefly the objective is to determine if data from the scale satisfy the expectation of the measurement model, a parametric probabilistic version of Guttman Scaling [13]. Where data do satisfy the expectations the manifest raw score from the summated the set of items can be transformed into interval scale measurement [22].

The process involves a number of activities, which include testing to see if the data meet Rasch model expectations; information on the quality of individual items including individual item fit; testing the assumption of unidimensionality; checking to see if the scale works in the same way across groups (invariance as determined by differential item functioning) and examining the reliability and targeting of the scale to the sample.

Initially, for polytomous items, a test is undertaken to establish which version of the Rasch model is appropriate, the Rating Scale version [23] or the Unrestricted (partial credit) version of the scale [24]. Fit to the Rasch model is then tested, and is achieved when a summary chi-square interaction statistic is non-significant, showing no deviation from model expectation; where item and person summary fit statistics show a mean of zero and standard deviation of one; where individual items show non-significant chi-square fit statistics (Bonferroni adjusted), and where individual item and person residuals are within the range of ±2.5. In addition, the scale is expected to show invariance across key groups (e.g., gender, age and previous experience of acupuncture), as indicated by a non-significant ANOVA of the residuals where group is the main factor, and to demonstrate strict unidimensionality, as indicated by an independent *t*-test on separate estimates for each respondent where <5% of such tests should be significant (the separate estimates are derived from subsets of items identified by a principal component analysis of the residuals). Reliability indices are also calculated, namely, the Person Separation Index (PSI).

Bonferroni corrections were applied throughout the analysis to allow for multiple testing (*P* < .01) [25]. Mokken scale analysis was undertaken with procedure “msp” within STATA [26]. Rasch analysis was conducted using RUMM2020 software [27]. Factor analysis and all descriptive analyses were conducted using SPSS 15.0 [28].

## 3. Ethics

Ethics approval was gained from the Southampton and South West Hampshire and the Salisbury and South Wiltshire Research ethics Committees (approval number 170/03/t).

## 4. Results

Total 221 patients completed the study (mean age 66.8; in S.D. 8.29, 58% females and 42% males). Their median pain score measured with visual analog scales (median over 7 days before the commencement of the study) was 59.4 (IQR 48.0–68.9). Of the total patients, 29% had previous experience of acupuncture.  Table 2 displays the distribution of scores on the HCAMQ items.

### 4.1. HCAMQ Scale Structure

**Figure 1:**
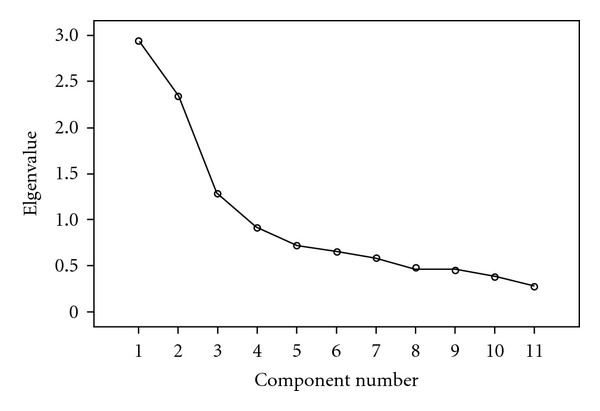
Scree Plot of the 11 items of the HCAMQ.

Both factor analysis and Mokken scaling failed to support the original two-factor structure of the scale. Three significant factors, where eigenvalues exceeded the Monte Carlo simulated values in parallel analysis, showed 10 of the items loading significantly on three subscales, and one item cross loading. The three-factor solution explained 60% of the variance in the data (first factor 27%, second factor 21%, third factor 12%, Table 3, Figure 1). The fourth eigenvalue was 0.910 and the associate parallel analysis value was 1.11, so rendering the fourth factor non-significant. The pattern of 10 items across 3 subscales was confirmed by the Mokken scaling, a negatively phrased CAM subscale (Loevinger 0.474); a positively phrased CAM subscale (Loevinger 0.379) and a Health Beliefs subscale (Loevinger 0.559).

### 4.2. Rasch Analysis of the Original HH Beliefs Questionnaire

**Figure 2:**
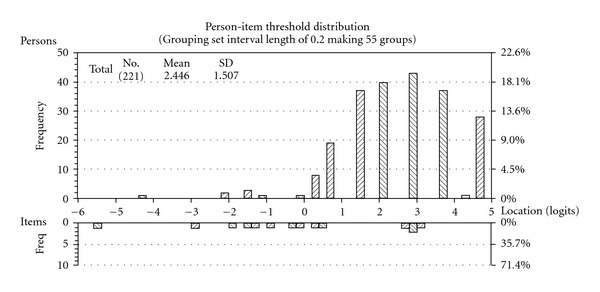
Person-item threshold map of the four-item Holistic Health Beliefs subscale.

The HH subscale items initially did not fit the Rasch model, as indicated by a significant Chi-square value and unacceptable number of significant *t*-tests when examining unidimensionality (Table 4, analysis 1). All items but one had disordered thresholds. That is, the transition between adjacent categories within an item did not reflect an increase in the underlying trait. To address this problem a number of strategies were considered. Initially, responses of all items were collapsed in identical ways to explore if this would achieve ordered thresholds (Table 4, analyses 2–4). However, combining responses “mildly agree” with “mildly disagree” (analysis 2); combining “mildly agree” with “agree” and “mildly disagree” with “disagree” (analysis 3); or combining “strongly agree” with “agree” and “strongly disagree” with “disagree” (analysis 4) did not result in ordered thresholds. Consequently items were rescored on an individual basis which resulted in ordered thresholds, and acceptable fit statistics model (non-significant chi-squares; fit residuals within the range –2.5 to +2.5) (Table 4, analysis 5). No DIF was found for any of the items. PSI remained at 0.69. However, the residual correlation matrix did show some correlations >0.30, in particular in relation to item 7. In addition, the Principal Component Analysis (PCA) of the residuals revealed that item 7 gave an extremely high positive loading on the first residual factor, suggesting that it may be problematic in this construct. Therefore a solution was sought by deleting item 7 (Table 4, analysis 6). After rescoring the data did not significantly deviate from the Rasch model expectation, and met the assumption of unidimensionality. All items were shown to fit the model, and the PSI remained at 0.69. The Person Item Threshold map shows the distribution of item thresholds and participants, demonstrating that many people had pro-HH beliefs (Figure 2).

### 4.3. CAM Beliefs Subscale

The CAM subscale was found to deviate significantly from the Rasch model (Table 5, analysis 1). Again most item thresholds were disordered, but addressing this issue did not result in a satisfactory fit to the model (Table 5, analysis 2–4). Once the items were ordered, closer inspection of the item fit showed that two items had significant misfit (significant chi-squares *P* < .01, Items 8 and 11, both positively worded), and item 11 had a high positive residual (2.679). Deleting these two items from the subscale resulted in a satisfactory, unidimensional four-item scale, with a PSI of 0.73 (Table 5, analysis 5).

**Figure 3:**
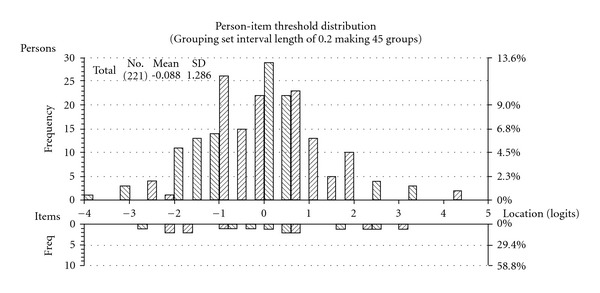
Person-item threshold map of the four-item CAM Beliefs subscale (negatively worded items).

Given this solution was consistent with both the factor analysis and Mokken scaling above, two new subscales were created, the first comprising of four items (negative CAM) and the second two items (positive CAM), reflecting the negative and positive directions of the item sets. After appropriate rescoring, both these new scales fitted the Rasch model (Table 5, analysis 6–7). The PSI of the first scale is 0.77 suggesting it can discriminate between three discrete groups. The Person Item Threshold map of this four-item scale is shown in Figure 3, demonstrating a good spread of item thresholds and people along the continuum. The second scale had a PSI of 0.51 which is not satisfactory.

## 5. Discussion

Health beliefs and attitudes to traditional and complementary medicine are increasingly acknowledged to have an important potential mediating role in behaviors and outcomes [29–32]. Thus, the ability to measure such constructs is also seen as increasingly important [33]. This study has examined one such potential scale, the HCAMQ from a largely modern psychometric perspective. Using three different approaches to testing unidimensionality all gave the same solution in that the original structure could not be supported and that a total HCAMQ score is not viable. Although we were unable to compare our eigenvalues with previous research, this finding is somewhat inconsistent with conclusions by others [9, 10]. While the HH Beliefs scale fitted the model (after removing one item), the CAM scale required splitting into two subscales, reflecting item sets which are worded in a positive and negative way. This is not unusual, as positive-negative orientation has been shown to give rise to different dimensions in that respondents seem to perceive such item sets in a different way, rather than just a “flipping over” of the item responses [34]. The second subscale, with just two items and explaining only 12% of the variance, is not viable. There always is a tension between the requirements of measurement and the need for content validity. Each looks at a slightly different aspect, the former that it is valid to add together a set of items, the latter that the scale is measuring appropriate content. The skill is to satisfy the former without compromising the latter. We therefore propose that the developers may wish to consider re-wording these two items in a negative frame in order to complement the other four items in the original subscale. Further testing of the revised scale would be needed to examine content and internal validity of the revised subscale.

We chose the Rasch model for this analysis because of its particular properties associated with fundamental measurement, specifically, the raw score as a sufficient statistic, and the separation of person and item parameters. The former is important as most everyday use is where clinicians and others add up the set of responses to make a total score, and thus the requirement is for the raw score to be a sufficient statistic. Secondly, quite often change scores and other mathematical operations are required of the data and the Rasch model is the only such Item Response Theory model that provides an interval scale transformation of the data.

The complementary use of factor analysis, Mokken scaling and Rasch analysis allows for a more thorough investigation of scaling properties than otherwise might be the case. Rasch analysis places considerable demands upon items sets, as it seeks to satisfy the basic axioms of constructing interval scale measurement [35]. On the other hand, Mokken scaling will determine if an ordinal scale has been constructed, which may be all that is needed, although this would restrain analysis to specific procedures, and could not support the calculation of change scores [36]. However, this would be perfectly appropriate where scales are used, for example, with cut scores, which just require a magnitude of the construct under investigation. In this study however, the Mokken analysis did not support the two HCAMQ subscales.

The study included people with OA of the knee and hip, waiting for a joint replacement. In Rasch analysis, item difficulty is calculated independently of the distributions of persons responding to the items. This “specific objectivity” is consistent with fundamental measurement and is only available for the family of Rasch models. However, many participants in our study displayed positive attitudes in particular toward holistic health although that could be due to self-selection bias into a study specifically looking at the effectiveness of acupuncture. Further, 29% had previous experience of acupuncture. The use of CAM is not uncommon in people with chronic musculoskeletal pain as their pain is often non-responsive to conventional primary care treatments and others have also reported high usage of CAM [37–40]. Nevertheless, for future validation studies of the HCAMQ it would be useful to include other groups of patients to ensure the scale is tested at both ends of the latent construct.

In conclusion we have shown that the original two-factor structure of the HCAMQ could not be supported and that a total HCAMQ score is not viable. Two valid shortened subscales can be used, one for HH Beliefs (four-item HH), and the other for CAM Beliefs (four-item CAM). It is recommended that consideration is given to rewording the two discarded positively worded CAM questions to enhance construct validity.

## Funding

This project was funded by a Department of Health Post Doctorial Fellowship Award (NCC RCD-CAMs 03/12).

## Figures and Tables

**Table 1:** Holistic complementary and alternative medicines questionnaire items.

CAM Subscale	HH Subscale
Question 2	Question 1^a^
Complementary medicine should be subject to more scientific testing before it can be accepted by conventional doctors	Positive thinking can help you fight off a minor illness
Question 4	Question 3^a^
Complementary medicine can be dangerous in that it may prevent people getting proper treatment	When people are stressed it is important that they are careful about other aspects of their lifestyle (e.g., healthy eating) as their body already has enough to cope with
Question 6	Question 5^a^
Complementary medicine should only be used as a last resort when conventional medicine has nothing to offer	The symptoms of an illness can be made worse by depression
Question 8^a^	Question 7^a^
It is worthwhile trying complementary medicine before going to the doctor	If a person experiences a series of stressful life events they are likely to become ill
Question 9	Question 10^a^
Complementary medicine should only be used in minor ailments and not in the treatment of more serious illness	It is important to find a balance between work and relaxation in order to stay healthy
Question 11^a^	
Complementary medicine builds up the body's own defences, so leading to a permanent cure	

Response options to each item: (1) Strongly agree, (2) Agree, (3) Mildly agree, (4) Mildly disagree, (5) Disagree, (6) Strongly disagree.

^a^Reversed scores for questions 1, 3, 5, 7, 8, 10, 11; higher scores reflect pro-CAM and pro-HH beliefs.

**Table 2:** Distribution of item responses.

Items	Frequency of responses (%)						
	1 (%)	2 (%)	3 (%)	4 (%)	5 (%)	6 (%)	Not answered (%)
HH subscale							
1^a^	—	4 (1.8)	6 (2.7)	25 (11.3)	98 (44.3)	88 (39.8)	—
3^a^	2 (0.9)	3 (1.4)	3 (1.4)	19 (8.6)	103 (46.6)	91 (41.2)	—
5^a^	3 (1.4)	7 (3.2)	2 (0.9)	15 (6.8)	89 (40.3)	105 (47.5)	—
7^a^	—	20 (9.0)	20 (9.0)	61 (27.6)	85 (38.5)	35 (15.8)	—
10^a^	5 (2.3)	1 (0.5)	3 (1.4)	9 (4.1)	108 (48.9)	94 (42.5)	1 (0.5)
CAM subscale							
2	32 (14.5)	84 (38.0)	48 (21.7)	24 (10.9)	24 (10.9)	9 (4.1)	—
4	15 (6.8)	26 (11.8)	50 (22.6)	42 (19.0)	68 (30.8)	18 (8.1)	2 (0.9)
6	12 (5.4)	27 (12.2)	34 (15.4)	34 (15.4)	79 (35.7)	34 (15.4)	1 (0.5)
8^a^	11 (5.0)	51 (23.1)	39 (17.6)	57 (25.8)	52 (23.5)	10 (4.5)	1 (0.5)
9	20 (9.0)	28 (12.7)	41 (18.6)	47 (21.3)	74 (33.5)	11 (5.0)	—
11^a^	4 (1.8)	17 (7.7)	39 (17.6)	105 (47.5)	48 (21.7)	8 (3.6)	—

Response options to each item: (1) Strongly agree, (2) Agree, (3) Mildly agree, (4) Mildly disagree, (5) Disagree, (6) Strongly disagree.

^a^Raw scores shown here. For analyses purposes scores would be reversed.

**Table 3:** Factor loadings of the 11 items of the HCAMQ, including factor eigenvalue and parallel analysis values.

Item	Factor loading		
	1	2	3
5	0.801		
10	0.795		
3	0.795		
1	0.740		
7	0.399	−0.340	
6		0.833	
9		0.815	
4		0.754	
2		0.563	
8			0.848
11			0.747
Eigenvalue	2.935	2.338	1.279
Parallel analysis	1.373	1.256	1.185

**Table 4:** Holistic health beliefs subscale rasch analysis results.

Analysis number	Item fit residual		Person fit residual		*χ* ^2^ interaction		PSI	Unidimensionality independent *t*-test (%) (95% CI)
	Mean	S.D.	Mean	S.D.	Value (df)	*P*		
1	−0.327	1.401	−0.315	0.768	23.77 (10)	0.008	0.69	10.48 (7.5 to 13.4)
2	0.011	1.437	−0.321	0.968	22.25 (10)	0.014	0.69	2.86 (−0.1 to 5.5)
3	−0.242	1.230	−0.320	0.922	24.88 (10)	0.006	0.66	2.86 (−0.1 to 5.5)
4	−1.428	0.974	−0.451	0.555	20.45 (5)	0.001	0.52	0 (−3.8 to 3.8)
5^a^	0.492	1.035	−0.293	1.031	20.46 (10)	0.025	0.69	2.38 (−0.6 to 5.3)
6^b^	0.357	0.431	−0.294	0.957	8.44 (8)	0.392	0.69	1.56 (−1.5 to 4.6)

^a^All items have been satisfactorily rescored so that thresholds are ordered; ^b^Results after the removal of item 7.

**Table 5:** Complementary and Alternative Medicine beliefs subscale Rasch analysis results.

Analysis number	Item fit residual		Person fit residual		*χ* ^2^ interaction		PSI	Unidimensionality independent *t*-test (%) (95% CI)
	Mean	S.D.	Mean	S.D.	Value (df)	*P*		
1	0.278	2.097	−0.370	1.227	62.78 (12)	<.001	0.68	7.24 (4.4 to 10.1)
2	−0.331	1.818	−0.554	1.361	57.48 (12)	<.001	0.65	12.22 (9.3 to 15.1)
3	−0.425	1.967	−0.487	1.181	40.86 (12)	<.001	0.64	7.24 (4.4 to 10.1)
4	−0.228	2.112	−0.430	1.145	52.82 (12)	<.001	0.69	7.24 (4.4 to 10.1)
5^a^	−0.289	1.408	−0.498	1.018	6.90 (8)	.55	0.73	3.21 (0.3 to 6.1)
6^b^	0.465	0.824	−0.615	1.552	17.33 (8)	.03	0.77	4.59 (1.7 to 7.5)
7^c^	0.232	1.789	−0.445	0.797	8.89 (4)	.06	0.51	1.38 (−1.5 to 4.3)

^a^In  analysis 3, questions 8 and 11 have been deleted, thus a 4-item subscale remains; ^b^Analysis 6 gives the results for the 4-item CAM scale (questions 2, 4, 6 and 9); ^c^Analysis 7 gives the results for the 2-item CAM scale (questions 8 and 11).
